# Relational Persistent Homology for Multispecies Data with Application to the Tumor Microenvironment

**DOI:** 10.1007/s11538-024-01353-6

**Published:** 2024-09-17

**Authors:** Bernadette J. Stolz, Jagdeep Dhesi, Joshua A. Bull, Heather A. Harrington, Helen M. Byrne, Iris H. R. Yoon

**Affiliations:** 1 Laboratory for Topology and Neuroscience, EPFL, Station 8, Lausanne, 1015 Switzerland; 2 Mathematical Institute, University of Oxford, Andrew Wiles Building, Woodstock Rd, Oxford, OX2 6GG UK; 3 Wellcome Centre for Human Genetics, University of Oxford, Roosevelt Dr, Headington, Headington, Oxford, OX3 7BN UK; 4 Ludwig Institute for Cancer Research, University of Oxford, Old Road Campus Research Build, Roosevelt Dr, Headington, Oxford, OX3 7DQ UK; 5 Department of Mathematics and Computer Science, Wesleyan University, 265 Church Street, Middletown, 06459 USA

**Keywords:** Persistent homology, Dowker complex, Witness complex, Multiplex imaging, Spatial relations, 62R40, 55N31, 62P10

## Abstract

Topological data analysis (TDA) is an active field of mathematics for quantifying shape in complex data. Standard methods in TDA such as persistent homology (PH) are typically focused on the analysis of data consisting of a single entity (e.g., cells or molecular species). However, state-of-the-art data collection techniques now generate exquisitely detailed multispecies data, prompting a need for methods that can examine and quantify the relations among them. Such heterogeneous data types arise in many contexts, ranging from biomedical imaging, geospatial analysis, to species ecology. Here, we propose two methods for encoding spatial relations among different data types that are based on Dowker complexes and Witness complexes. We apply the methods to synthetic multispecies data of a tumor microenvironment and analyze topological features that capture relations between different cell types, e.g., blood vessels, macrophages, tumor cells, and necrotic cells. We demonstrate that relational topological features can extract biological insight, including the dominant immune cell phenotype (an important predictor of patient prognosis) and the parameter regimes of a data-generating model. The methods provide a quantitative perspective on the relational analysis of multispecies spatial data, overcome the limits of traditional PH, and are readily computable.

**Supplementary Information:**

The online version contains supplementary material available at 10.1007/s11538-024-01353-6.

## Introduction

Topological data analysis (TDA) is a field of mathematics that develops topological tools for detecting the shape of data. A prominent tool in TDA, persistent homology (PH) (Ghrist 2008; Edelsbrunner and Harer 2008; Carlsson 2009; Edelsbrunner et al 2000), constructs a nested sequence of topological scaffolds of shapes from data, called a filtration of simplicial complexes. PH examines the evolution of topological features such as connected components (dimension 0) and loops (dimension 1) across the filtration. The filtration is constructed from meaningful aspects of the data at multiple scales such as distances (Vietoris 1927; Edelsbrunner 1993), function values (Chazal et al 2013; Günther et al 2012), and densities (Carlsson and Zomorodian 2007; Botnan and Lesnick 2023; Vipond et al 2021). One possible input to PH is point cloud data, and the output is a persistence diagram, which can be vectorized and integrated with statistics and machine learning methods (Ali et al 2022). PH provides an automatic, robust, and interpretable method for analyzing data arising in many fields of biology and medicine, including cancer biology (Lawson et al 2019; Singh et al 2014; Chittajallu et al 2018; Aukerman et al 2020; Nicolau et al 2011; Bhaskar et al 2021; Nardini et al 2021; Stolz et al 2020; Yang et al 2023), neuroscience (Gardner et al 2022; Curto and Itskov 2008; Dabaghian et al 2012; Giusti et al 2015), and genomics (Masoomy et al 2021; Cámara 2017; Benjamin et al 2023; Emmett et al 2015; Chan et al 2013).

Most existing PH applications are limited to the study of data relating to one species. Advanced data collection techniques now generate multispecies data in which distinct species may interact. Data of this nature are ubiquitous in science, ranging from cancer biology and ecology to geospatial analysis. By studying the spatial relationships among species, we can glean insights that would otherwise be missed in non-spatial analyses. Extracting spatial relationship information from such data, therefore, requires the development of novel analysis techniques. Recently, two topological methods have been proposed to study multispecies data (Bhaskar et al 2022; di Montesano et al 2022). The first approach concatenates topological features from different cell types in cancer images (Bhaskar et al 2022) but does not capture spatial relations between the different cell types. Another method, the chromatic alpha complex (di Montesano et al 2022), encompasses relations among species by constructing a multispecies version of the Delaunay triangulation; its computational implementation and interpretation are still under development.

Here, we present two topological approaches for encoding spatial relations among different species directly at the input level for PH. We implement and showcase these methods on synthetic multispecies data generated by an agent-based model (ABM) of the tumor microenvironment. We present two example pipelines incorporating the two topological encodings of spatial relations. The pipelines can be adjusted by making different choices for the topological approaches and their combination with vectorizations. We show that topological relations encode biological insight by predicting the dominant immune cell phenotype and by clustering the parameter regimes of the data-generating model using the relational topological features.

Mathematically, the multispecies data we consider can be viewed as a labeled point cloud $P = \bigcup _{i=0}^{m} P_i$ that consists of $m+1$ different species whose spatial distributions may be related to one another. Each point $p \in P$ is in $\mathbb {R}^2$. Note that both topological methods can be applied to point clouds in $\mathbb {R}^n$ for $n \ge 2$. We generated synthetic multispecies spatial data from an ABM that simulates the behavior of different cell types in a tumor microenvironment (Bull and Byrne 2023). The proposed topological methods are built on Dowker complexes (Dowker 1952) and witness complexes (de Silva and Carlsson 2004). These *relational PH* methods, which we refer to as *Dowker PH* and *multispecies witness PH*, use one species, e.g., $P_0$, as the potential vertex set for a simplicial complex and use another species to create a filtration.

Dowker PH (Chowdhury and Mémoli 2018) is based on a Dowker complex (Dowker 1952), which is a simplicial complex that represents relations between two point clouds. Dowker complexes have been used to capture relations in molecular biology (Liu et al 2022), networks (Chowdhury and Mémoli 2018), PDF parsers (Ewing and Robinson 2021), and persistence diagrams (Yoon et al 2023). We propose using Dowker PH (Chowdhury and Mémoli 2018), a natural extension of Dowker complexes, for multispecies data. Dowker PH of the pair $(P_i, P_j)$ creates a filtered Dowker complex on points $P_i$ based on proximity to points in $P_j$. Dowker PH then examines the topological features of the Dowker complex that evolve as one varies the distances between $P_i$ and $P_j$. The resulting Dowker persistence diagram is agnostic to the choice of $P_i$ or $P_j$ as the vertex set and can informally be interpreted as capturing shared topological features, i.e., connected components and loops, between $P_i$ and $P_j$.

While Dowker PH encodes pairwise relations, it does not capture how one species, say $P_0$, relates to all other species in *P*. To capture differences between all relations among every pair $(P_0, P_i)$, we present a second approach called *multispecies witness PH*, which is inspired by the lazy witness filtration (de Silva and Carlsson 2004). The multispecies witness filtration first creates a Delaunay triangulation (Delaunay 1934) on $P_0$ and creates a filtration based on the number of points in $P_i$ close to simplices in $P_0$. We chose the Delaunay triangulation because of its simplicity and close relationship to the lazy witness filtration (see Theorem 3 in de Silva and Carlsson (2004))^1^. To encode $P_0$’s different spatial relations to all other subpopulations, we construct *m* separate filtrations, measure the distance between their topological features, and combine these distances into a *topological distance vector*, which can then be used as input into classification or machine learning tools.

We apply Dowker PH and multispecies witness PH to two different, yet related biological problems arising from the data-generating model to showcase variations of relational PH. In both application pipelines, we combine the PH methods with vectorizations that emphasize relevant aspects of the encoded relations for the studied problem. In the first problem, we investigate the prediction of the dominant macrophage phenotype, and we use persistence images to vectorize the spatial interactions captured by the Dowker features. Because the dominant macrophage phenotype changes over time, we analyze data simulated at different time points. In the second problem, we classify qualitative regimes of the model which are determined by differences in relative spatial locations using multispecies witness PH. Here, we use distance vectors to detect differences between the cell type-dependent filtrations that we create. The qualitative regimes are classified at the final time point of the simulation (Bull and Byrne 2023), so we analyze the point clouds at the final time point of the simulation.

The paper is organized as follows. In Sect. 2, we describe the synthetic multispecies data and introduce the two questions arising in the study of data from the tumor microenvironment. In Sect. 3, we briefly review the mathematical preliminaries of PH. In Sect. 4, we present the relational PH approaches designed for capturing relations among multiple species: Dowker PH and multispecies witness PH. In Sect. 5, we showcase these methods on a simulated tumor microenvironment and address the biologically motivated questions introduced in Sect. 2. The paper concludes in Sect. 6 where we discuss our results and outline directions for future research.

## Multispecies Spatial Data

We introduce the data set we later analyze, which is synthetic point clouds of multiple species in a simulated tumor microenvironment. Next, we state the two associated domain-specific questions that motivate this mathematical study.

### Point Clouds Simulated Via Agent-Based Modeling

We study point clouds representing a dynamic and spatially-resolved tumor microenvironment generated by an agent-based model (ABM). ABMs simulate the emergent behavior of a system through the enactment of rules that determine the outcome of interactions between their constituent ‘agents’, here typically individual cells (Bonabeau 2002). They are ideally suited to create multispecies data. We use the ABM presented by Bull and Byrne (2023). See SI Section 1 and Bull and Byrne (2023) for details.

Each simulation produces a point cloud *P* consisting of five species $P = P_T \cup P_S \cup P_N \cup P_M \cup P_V$. Each labeled point cloud represents the locations of tumor cells ($P_T$), stromal cells ($P_S$), necrotic cells ($P_N$), macrophages ($P_M$), and blood vessels ($P_V$). The spatial locations of the blood vessels are randomized at the start of each simulation and then held fixed. By contrast, all other cell types are assumed to be motile. Their movement is determined by interactions among the cells and five different diffusible species (oxygen, CSF-1, TGF-$\beta$, CXCL12, and EGF). We focus on simulations that arise by varying two key parameters of the model that affect the behavior of macrophages: $\chi ^m_c$, the chemotactic sensitivity of macrophages to spatial gradients of one of the chemical species (CSF-1), and $c_{1/2}$, a parameter regulating the rate at which macrophage extravasate from the blood vessels (Bull and Byrne 2023). We consider 9 different values for each parameter. For each of the 81 possible parameter pairs $(\chi ^m_c, c_{1/2})$, we generate up to 20 realizations of the ABM in which the positions of the blood vessels are varied.^2^ For each simulation, the point clouds are generated at 6 time points ($t = 250, 300, 350, 400, 450, 500$ hours). We focus on the behaviors of macrophages and tumor cells. Each macrophage has an associated phenotype, $\Omega \in [0,1]$, which determines how it interacts with tumor cells. Macrophages with low $\Omega$ have high tumor-killing capacity. Those with high $\Omega$ assist the migration of tumor cells towards the vasculature, thereby promoting metastasis. We refer to macrophages with phenotype $0 \le \Omega < 0.5$ as $M_1$ or anti-tumor macrophages; we refer to those with phenotype $0.5 \le \Omega \le 1$ as $M_2$ or pro-tumor macrophages. The cutoff value of 0.5 is motivated by the model described in Bull and Byrne (2023) in which $\Omega = 0.5$ describes the tipping point of the macrophage behavior. For $\Omega < 0.5$, the model exhibits anti-tumor behavior, whereas for $\Omega > 0.5$, the model is skewed in a pro-tumor direction.

Simulations are initially seeded with a small cluster of tumor cells at the center of the domain, with blood vessels clustered around the edge. Blood vessels act as sources of oxygen, which is consumed by both stromal cells and tumor cells. Tumor cells are sources of CSF-1, which diffuses through the domain and acts as a stimulus for the recruitment of macrophages and as a chemoattractant for them. During each simulation, macrophages with phenotype $\Omega =0$ enter the domain at a rate determined by CSF-1 levels at the blood vessels, with higher CSF-1 increasing the rate of macrophage extravasation. As a macrophage migrates through the domain, its phenotype changes in response to local levels of the different chemical species, including TGF-$\beta$. (For details, see SI Section 1).

For a given parameter set, at the end of each simulation ($t=500$ hours), we observe one of three distinct qualitative behaviors:

- **tumor elimination**, in which $M_1$ macrophages dominate the simulation and the tumor cells have been eliminated;
- **tumor equilibrium**, in which macrophages are unable to eliminate the tumor cells which form a compact mass, surrounded by macrophages that are predominantly of an $M_1$ phenotype;
- **tumor escape**, in which $M_2$ macrophages enhance tumor cell migration to the vasculature. These simulations are characterized by the formation of perivascular niches in which $M_2$ macrophages, tumor cells, and blood vessels are found in close proximity. Such behavior is associated with metastasis of tumor cells (Arwert et al 2018).

We consider two subsets of data generated by the ABM. The first data subset is generated from 2 realizations of $9\times 9$ parameter combinations of $c_{1/2}$ and $\mathcal {X}_c^m$. The point clouds are generated at 6 time points ($t = 250, 300, 350, 400, 450, 500$ hours) of the simulation, resulting in $972 = 6 \times 2 \times 9 \times 9$ point clouds. We use the first data subset to predict the dominant macrophage phenotype. All simulations at various time points are analyzed because the dominant macrophage phenotype changes over time.

For the second data subset, we consider up to 20 realizations of $9\times 9$ parameter combinations of $c_{1/2}$ and $\mathcal {X}_c^m$, i.e., a maximum of 1620 point clouds. As noted by Bull and Byrne (2023), limitations on HPC time meant that for some parameter combinations, fewer than 20 realizations were available, giving a total of 1485 point clouds generated at a single ‘endpoint’ time ($t = 500$ hours). For each point cloud, we use the positions of tumor cells, blood vessels, and macrophages (with and without knowledge of macrophage phenotype) as input. For comparison, we also construct simple, i.e., non-topological, descriptor vectors with entries corresponding to the number of tumor cells, the number of macrophages, the number of necrotic cells, the average distance of tumor cells to the nearest blood vessel, the average distance of necrotic cells to the nearest blood vessel, and the average distance of macrophages to the nearest blood vessel. We use the second data subset to classify the parameter regimes that lead to different qualitative behaviors. The qualitative behaviors are labeled at the final time point of the simulation Bull and Byrne (2023). We therefore analyze the point clouds generated at the final time point.

### Statement of Biologically Motivated Problems

**Fig. 1:**
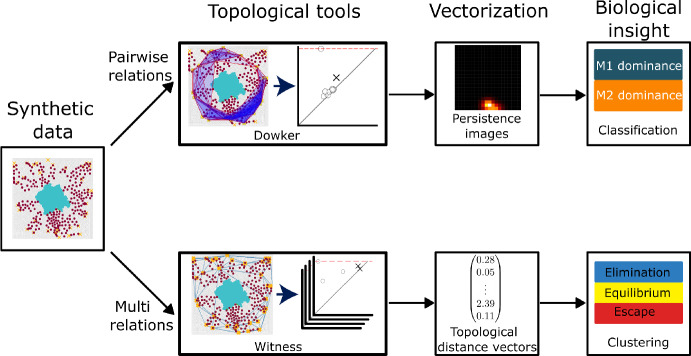
Relational PH pipeline and analysis. We use point clouds generated by an ABM as input to two different topological methods for encoding relations: Dowker PH (top row) and multispecies witness PH (bottom row). We vectorize Dowker topological descriptors using persistence images and vectorize witness topological descriptors via distances between them. Finally, we perform supervised binary classification to predict the dominant macrophage phenotype using Dowker features and perform unsupervised clustering to infer the parameter regimes of elimination, equilibrium, and escape using multispecies witness features

We address the following two biologically motivated questions regarding macrophage and tumor behavior:

1. Can relational PH predict the dominant macrophage phenotype from the cell locations without knowledge of the phenotypes of individual macrophages?
2. Can relational PH identify the parameter regimes of the ABM that lead to different qualitative behaviors: tumor elimination, escape, and equilibrium with macrophages?

These two questions motivated the two pipelines shown in Fig. 1, with the first problem corresponding to the pipeline in the top row and the second to the pipeline introduced in the bottom row. While we present pipelines that were effective in this study, the pipelines may be adjusted by making different choices for the topological tools and vectorizations (see also Sect. 6).

#### Problem 1: Prediction of Dominant Macrophage Phenotype

**Fig. 2:**
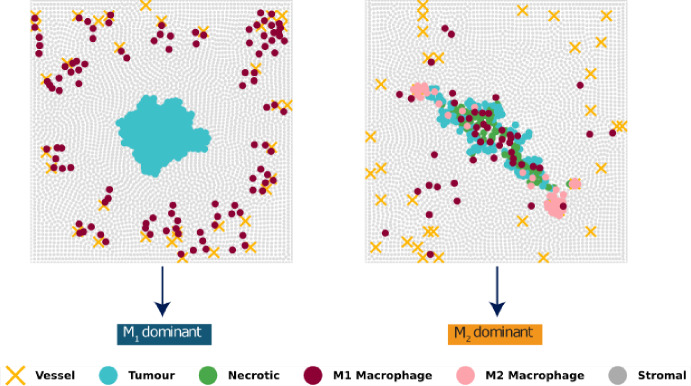
Problem 1: Prediction of dominant macrophage phenotype. Given a simulated tumor microenvironment, can we predict the dominant macrophage phenotype?

We examine whether relational features can predict the dominance of $M_1$ and $M_2$ macrophages (see Fig. 2), which is an important predictor of a cancer patient’s overall survival time (Jayasingam et al 2020). Macrophage phenotype prediction problems may arise in experimental and clinical settings when analyzing imaging data that contains a single macrophage marker or when conventional time- and resource-intensive methods of characterizing macrophage phenotype are not viable (Rostam et al 2017; Huang et al 2019; Jayasingam et al 2020; Misharin et al 2013). We use Dowker PH for this task due to its pairwise encoding of relations. Dowker’s shared topological features allow biological interpretation of which relative cell locations directly influence macrophage phenotype. In particular, since macrophage phenotype is influenced by its spatial interactions with blood vessels and tumor cells, Dowker PH is ideally suited to quantify these interactions. In subsequent analysis, we use persistence images as a vectorization to retain the pairwise relations captured by the Dowker persistence diagrams. We demonstrate that relational PH can identify the dominant macrophage phenotype based on the spatial relations among the constituents.

#### Problem 2: Classification of Parameter Regimes Leading to Different Qualitative Behaviors of the ABM

**Fig. 3:**
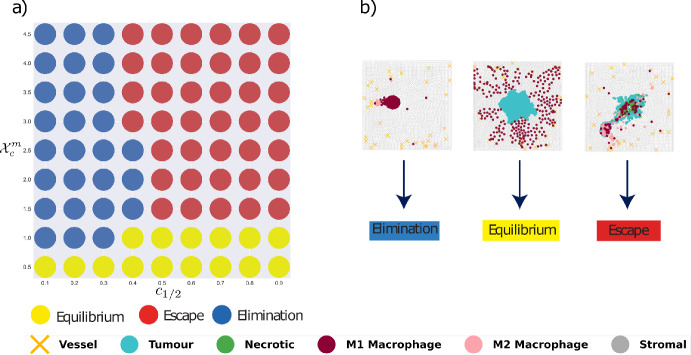
Problem 2: Classification of parameter regimes leading to different qualitative behaviors of the ABM. **a** Parameter values of $c_{1/2}$ and $\mathcal {X}_c^m$ varied in the ABM. Depending on the parameter combination, a simulation of the tumor microenvironment results in one of three qualitative behaviors: elimination of the tumor (blue), equilibrium of tumor cells and macrophages (yellow), and escape of the tumor cells towards blood vessels (red). The parameter combinations are colored according to the subjective classification of the qualitative behavior observed in one simulation of the model. **b** Can we systematically determine the different qualitative behaviors of the ABM from the locations of the different cell types? (Color figure online)

Secondly, we explore the use of relational PH in understanding the parameter regimes used to generate different simulations, specifically to classify different parameter regimes from the spatial distribution of the different cell types (see Fig. 3). The ABM parameters influence the spatial distributions of different cell types in the tumor microenvironment, leading to different tumor compositions and morphology. The qualitative behaviors that arise from the different parameter combinations of the ABM are shown in Fig. 3 a). The qualitative behaviors were subjectively assigned in the paper (Bull and Byrne 2023). Capturing these differences objectively from the spatial patterns of cells could pave the way for the automated identification of disease stages in microscopy images. Since we are interested in classifying long-term tumor outcomes (escape, elimination, and equilibrium), we consider the ABM output at a single late ‘endpoint’ time ($t = 500$ hours) for varying combinations of parameters $c_{1/2}$ and $\mathcal {X}_c^m$. Multispecies witness PH is ideally suited to this task since it simultaneously takes into account all species in the data set and focuses on their relative spatial locations. When combined with distance computations, it can be used to highlight differences in relative spatial distributions.

## Mathematical Preliminaries

We briefly introduce the standard PH, which can be used to analyze the spatial patterns of point cloud data. For details of PH, see Ghrist (2008); Edelsbrunner and Harer (2008); Carlsson (2009); Edelsbrunner et al (2000).

### Persistent Homology

Let *P* denote a point cloud of data in $\mathbb {R}^n$. Here, *P* is a point cloud of data in $\mathbb {R}^2$ describing the spatial location of biological cells such as cancer cells. The spatial patterns and structure of *P* can be studied by constructing filtered simplicial complexes, i.e., collections of vertices, edges, triangles, and their higher-order counterparts that can be glued together to approximate topological spaces. We refer to each building block as a simplex. A 0-simplex is a single point in *P*, a 1-simplex is an edge between two points in *P*, a 2-simplex is a triangle among three points, and so on. We denote an *n*-simplex by the collection of $n+1$ vertices $(p_0, \dots , p_n)$ that are involved. The standard choice of a filtered simplicial complex is the Vietoris-Rips filtration $\mathcal{V}\mathcal{R}_P$ (Vietoris 1927):

#### Definition 1

(Vietoris-Rips filtration) Let *P* be a point cloud and let *d* be a distance on *P*. The *Vietoris-Rips complex at parameter* $\varepsilon$, denoted $\mathcal{V}\mathcal{R}_P^{\varepsilon }$, is an abstract simplicial complex that has *P* as the vertex set and has the *n*-simplex $\sigma = (p_0, \dots , p_n)$ if $d(p_i, p_j) \le \varepsilon$ for all $p_i, p_j \in \sigma$. A *Vietoris-Rips filtration* $\mathcal{V}\mathcal{R}_P^{\bullet }$ is a nested sequence of simplicial complexes $\mathcal{V}\mathcal{R}_P^{\varepsilon }$ for varying $\varepsilon$.

**Fig. 4:**
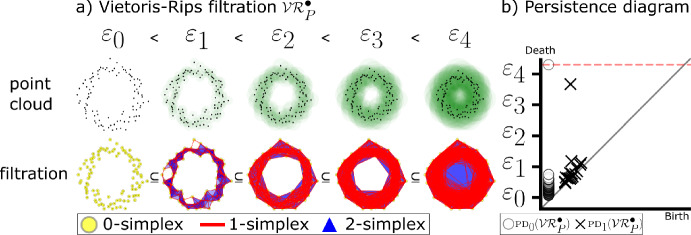
An example Vietoris-Rips filtration. **a** Example Vietoris-Rips complexes $\mathcal{V}\mathcal{R}_P^{\varepsilon }$ at various $\varepsilon$ parameters. The top row shows the point cloud (in black) and $\varepsilon /2$-ball neighborhoods around each point (in green) for varying $\varepsilon$ values. The bottom row shows the Vietoris-Rips filtration. For a fixed $\varepsilon$, we draw a 0-simplex for each point in the point cloud. Whenever the green balls intersect, we place a 1-simplex between the two corresponding points. We then fill in any higher-dimensional simplices that arise. **b** A persistence diagram provides a visual summary of the evolution of connected components (dimension 0, denoted $\text {pd}_0$) and loops (dimension 1, denoted $\text {pd}_1$). We show an overlay of the dimension-0 persistence diagram $\text {pd}_0(\mathcal{V}\mathcal{R}^\bullet _P)$ (in circle) and dimension-1 persistence diagram $\text {pd}_1(\mathcal{V}\mathcal{R}^\bullet _P)$(in cross). A point on the persistence diagram represents a topological feature. The *x*-coordinate is the parameter $\varepsilon$ at which the feature is born, and the *y*-coordinate is the parameter at which the feature dies. In $\text {pd}_0$, all connected components share the same birth parameter, and the death of a component occurs when two components merge. The red line indicates an infinite death value. There is one point with an infinite death parameter, indicating that the Vietoris-Rips filtration has a single connected component that never vanishes as we increase $\varepsilon$. In $\text {pd}_1$, there is a single point far from the diagonal, indicating that there is one significant loop with a small birth parameter and large death parameter. The remaining points can be considered as noise (Color figure online)

The Vietoris-Rips complex $\mathcal{V}\mathcal{R}_P^\varepsilon$ at parameter $\varepsilon$ represents the connectivity of *P* up to proximity $\varepsilon$ (see Fig. 4a). The Vietoris-Rips filtration $\mathcal{V}\mathcal{R}_P^{\bullet }$ encodes the connectivity of the point cloud at various proximity parameters. PH provides the means to study topological features such as connected components ($H_0$) and cycles ($H_1$) across nested simplicial complexes. Throughout this paper, we fix the field $\mathbb {F} = \mathbb {Z}/2 \mathbb {Z}$.

#### Definition 2

(Persistent homology) Given a nested sequence of simplicial complexesthe *dimension-k persistent homology* of $X^\bullet$ is a collection of $\mathbb {F}$-vector spaces

$$\begin{aligned} PH_k(X^\bullet ) = H_k(X^{\varepsilon _1}; \mathbb {F}) \xrightarrow {\phi ^{\varepsilon _1}} H_k(X^{\varepsilon _2}; \mathbb {F}) \xrightarrow {\phi ^{\varepsilon _2}} \cdots \xrightarrow {\phi ^{\varepsilon _{N-1}}} H_k(X^{\varepsilon _N}; \mathbb {F}), \end{aligned}$$

with $\phi ^{\varepsilon }$ being the maps induced by $\iota ^{\varepsilon }$.

The evolution of structural features across a filtration is obtained via the structure theorem.

#### Theorem 1

(Structure Theorem for persistent homology (Carlsson and Zomorodian 2005)) Any dimension-*k* persistent homology $PH_k(X^\bullet )$ obtained from a finite filtered simplicial complex $X^\bullet$ decomposes uniquely as

$$\begin{aligned} PH_k(X^\bullet ) \cong \bigoplus _{i} I_{b_i, d_i},\end{aligned}$$

where each $I_{b_i, d_i}$, called *an interval module*, is a sequence of $\mathbb {F}$-vector spaces

$$\begin{aligned} I_{b_i, d_i} = 0 \xrightarrow {\phi ^0} \cdots \xrightarrow {\phi ^{b_i-1}} \mathbb {F} \xrightarrow {\phi ^{b_i}} \cdots \xrightarrow {\phi ^{d_i-1}} \mathbb {F} \xrightarrow {\phi ^{d_i}} 0 \xrightarrow {\phi ^{d_i + 1}} \cdots \xrightarrow {\phi ^{N-1}} 0 \end{aligned}$$

with $\phi ^{\varepsilon }$ as identity maps for $\varepsilon \in [b_i, d_i)$ and zero otherwise.

Given an interval module $I_{b_i, d_i}$, the parameters $b_i$ and $d_i$ are referred to as the *birth* and *death* times of $I_{b_i, d_i}$. The length (death - birth) is referred to as *persistence*. The decomposition of $PH_k(X^\bullet )$ is often represented using the collection of birth and death times, and they are visualized using a persistence diagram (see Fig. 4b). We denote the dimension-*k* persistence diagram by $\text {pd}_k(X^\bullet ).$

Persistence diagrams are stable (Chazal et al 2009). That is, there exist distances on persistence diagrams such that small perturbations of the input *P* result in small changes in the persistence diagram. Two commonly used distances on persistence diagrams are the Wasserstein distance (Cohen-Steiner et al 2010) and the bottleneck distance (Cohen-Steiner et al 2005), which are described as follows.

#### Definition 3

Given two points $x = (x_b, x_d)$ and $y = (y_b, y_d)$ in a persistence diagram let $\Vert x - y \Vert _{\infty } = \max \{ |y_b - x_b |, |y_d - x_d |\}$. Given two persistence diagrams $\text {pd}_k(X^{\bullet })$ and $\text {pd}_k(Y^\bullet )$, the $q-$*Wasserstein distance* is

$$\begin{aligned} d_W(\text {pd}_k(X^\bullet ), \text {pd}_k(Y^\bullet )) = \inf _{\gamma : \text {pd}_k(X^\bullet ) \rightarrow \text {pd}_k(Y^\bullet )} \left( \sum _{x \in \text {pd}_k(X^\bullet )} \Vert x - \gamma (x) \Vert ^q_{2} \right) ^{1/q}, \end{aligned}$$

where $\Vert \cdot \Vert _{2}$ is the $L_2$ norm,^3^ and the *bottleneck distance* is

$$\begin{aligned} d_B(\text {pd}_k(X^\bullet ), \text {pd}_k(Y^\bullet )) = \inf _{\gamma : \text {pd}_k(X^\bullet ) \rightarrow \text {pd}_k(Y^\bullet )} \sup _{x \in \text {pd}_k(X^\bullet )} \Vert x - \gamma (x) \Vert _{\infty }. \end{aligned}$$

where $\gamma$ denotes a bijection between $\text {pd}_k(X^{\bullet })$ and $\text {pd}_k(Y^\bullet )$. In the case of cardinality mismatches between the persistence diagrams, points on the diagonal are included to generate this bijection.

In Sect. 5.2, we use the 1-Wasserstein distance and the bottleneck distance to construct entries of distance vectors between pairs of persistence diagrams. Both distances capture distinct differences between the two persistence diagrams that are compared. While the 1-Wasserstein distance takes into account how well all points between the two persistence diagrams agree and is additionally influenced by the number of points in a persistence diagram, the bottleneck distance focuses on the worst such agreement.

### Vectorization for Machine Learning

**Fig. 5:**
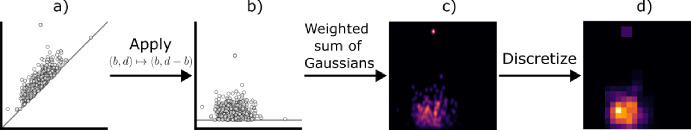
Vectorization of persistence diagrams via persistence image. **a** An example persistence diagram. **b** The result of mapping each point (birth, death) in a persistence diagram to (birth, death-birth). **c** A weighted sum of Gaussians centered at each point of **b**. **d** A discretized array of image **c**. The resulting persistence image is often flattened into a vector. Figure adapted from Adams et al (2017)

Given a persistence diagram $\text {pd}_k(X^\bullet )$, various techniques can be used to convert it into a vector that is compatible with standard statistics and machine learning (Ali et al 2022; Bubenik 2015). Here, we use persistence images (Adams et al 2017), which summarize the distribution of points on the persistence diagram using a weighted sum of Gaussian distributions centered at each point of the persistence diagram (see Fig. 5). A persistence diagram is first transformed by mapping each point $(\text {birth}, \text {death})$ to $(\text {birth}, \text {death - birth})$ (Fig. 5a,b). We then place a Gaussian distribution centered at each transformed point and assign a non-negative weighting function (Fig. 5b,c). The function places zero weight for points along the horizontal axis of Fig. 5b. The weighted sum of Gaussians is then discretized to produce an array called a persistence image (Fig. 5d). The persistence image is often flattened into a vector. The resulting vector is influenced by several parameters, including the width, $\sigma$ of the Gaussian, and discretization size. In this study, we use $\sigma = 1$ and discretize images to size $20\times 20$, resulting in flattened vectors of dimension 400.

## Introducing Filtrations for Multispecies Data

While standard PH detects structure in a point cloud, it fails to encode how multiple point clouds are related. We present two extensions of the standard PH pipeline to capture multi-system interactions: Dowker PH (Chowdhury and Mémoli 2018) and multispecies witness PH, a new construction motivated by witness complexes (de Silva and Carlsson 2004).

### Dowker Persistent Homology

Let *U* and *V* denote two distinct point clouds. In our study, *U* and *V* represent different biological cell types, such as tumor cells and macrophages. The structure of *U* from the viewpoint of *V* can be studied using a Dowker filtration:

**Fig. 6:**
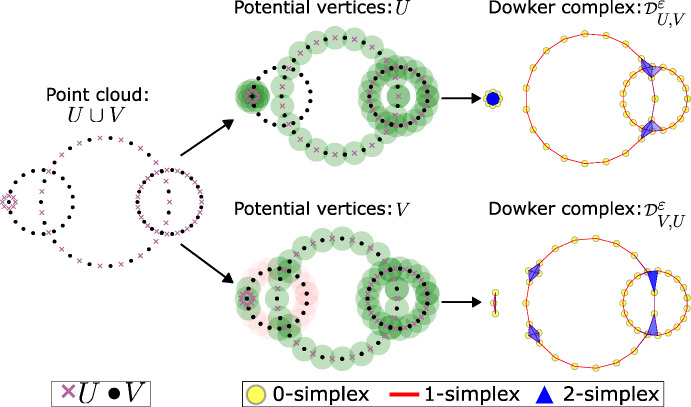
Example Dowker complexes. We present two Dowker complexes built on point clouds *U* and *V* for some proximity parameter $\varepsilon$. (Top) Dowker complex with *U* as the potential vertex set. (Bottom) Dowker complex with *V* as the potential vertex set. Given a potential vertex set, the $\varepsilon$-neighborhoods of the vertices are shown in green if the neighborhood contains an element of the other point cloud. Otherwise, the neighborhood is shown in red. A vertex with a green neighborhood becomes a 0-simplex in the Dowker complex. We add a 1-simplex between two vertices if their $\varepsilon$-neighborhood intersection contains a vertex from the other point cloud. We add a 2-simplex among three vertices if their $\varepsilon$-neighborhood intersection contains a vertex from the other point cloud (Color figure online)

#### Definition 4

(Dowker filtration (Dowker 1952; Chowdhury and Mémoli 2018)) Let *U* and *V* be point clouds, and let $d_{U,V}$ be the distance function between elements of *U* and *V*. A *Dowker complex at parameter* $\varepsilon$, denoted $\mathcal {D}_{U,V}^{\varepsilon }$, is a simplicial complex that has *U* as the potential vertex set and includes the *n*-simplex $\sigma = (u_0,\ldots ,u_n)$ if there exists a $v \in V$ such that $d_{U,V}(u_i,v) \le \varepsilon$ for all $u_i \in \sigma$. A *Dowker filtration* $\mathcal {D}_{U,V}^{\bullet }$ is a nested sequence of Dowker complexes $\mathcal {D}_{U,V}^{\varepsilon }$ for varying $\varepsilon$.

The Dowker complex $\mathcal {D}_{U,V}^{\varepsilon }$ at parameter $\varepsilon$ captures relations between *U* and *V*, where the relations are restricted to points (*u*, *v*) whose distance is at most $\varepsilon$. The Dowker complexes $\mathcal {D}_{U,V}^{\varepsilon }$ (Fig. 6, top) and $\mathcal {D}_{V,U}^{\varepsilon }$ (Fig. 6, bottom) each have *U* and *V* as the potential vertex set. Note that the two Dowker complexes resemble one another even though their vertex sets are distinct. For example, both Dowker complexes have two connected components and three 1-dimensional cycles, i.e., loops. Dowker’s Theorem states that the two Dowker complexes have the same homology groups, i.e., connected components and loops^4^ (Dowker 1952). In fact, the geometric realizations of the two Dowker complexes are homotopy equivalent (Björner 1996). Dowker complexes can capture shared topological features between two point clouds, as illustrated in Fig. 6. Note that there are instances in which the Dowker complex captures a feature present in *U* that isn’t present in *V*, for example, if *V* is a dense sample of a region containing *U* (see Sect. 5.1.2 for details).

To study the features of Dowker complexes across a range of parameters $\varepsilon$, we compute the PH of the Dowker filtration $\mathcal {D}_{U,V}^{\bullet }$. We call the resulting persistence diagram $\text {pd}_k(\mathcal {D}_{U,V}^{\bullet })$ the Dowker persistence diagram. The functorial Dowker’s Theorem states that the persistence diagrams of the two filtered Dowker complexes are the same.

#### Theorem 2

(Functorial Dowker’s Theorem (Chowdhury and Mémoli 2018)) $\text {pd}_k(\mathcal {D}_{U,V}^{\bullet }) = \text {pd}_k(\mathcal {D}_{V,U}^{\bullet })$ for all *k*.

The Dowker persistence diagram is a collection of birth and death parameters of *k*-dimensional topological features, i.e., connected components and loops for $k = 0$ and $k = 1$ respectively, in the Dowker filtration. In this study, we utilize dimension-0 and dimension-1 Dowker persistence diagrams. The Dowker persistence diagram can be vectorized via persistence images as described in Sect. 3 and then be used in various statistical and machine learning methods. Given points clouds of size *n* and *m* and a distance matrix of size $n \times m$, one can create the filtered Dowker complex as the following. For each column *j*, sort the column. The collection of rows whose $j^{\text {th}}$ entry is at most *p* represent a simplex that is present at filtration values *p* or higher. One can then go through the rows of the sorted $j^{\text {th}}$ column to create a collection of simplices that are present at various filtration values. Creating the filtered Dowker complex thus has a computational complexity of $\mathcal {O}(mn \log n)$.

### Multispecies Witness Persistent Homology

Our second approach is motivated by the construction of *(lazy) witness filtrations*. The (lazy) witness filtration was first introduced by de Silva and Carlsson (de Silva and Carlsson 2004) and has been used to study noisy artificial datasets (Kovacev-Nikolic 2012), primary visual cortex cell populations  (Singh et al 2008), and cancer gene expression data  (Lockwood and Krishnamoorthy 2015). Roughly, the lazy witness filtration is constructed via the following steps:

1. Select a subset of *landmark points* *L* from the point cloud *P*.
2. Construct a *lazy witness filtration* where the landmarks *L* are the vertex set and the full point cloud *P* serve as *witnesses* for higher order simplices. Broadly speaking, points in *P* are witnesses to the simplices on *L* to which they are closest. De Silva and Carlsson (de Silva and Carlsson 2004) demonstrate that the resulting simplicial complex can be interpreted as an intrinsic Delaunay triangulation (Delaunay 1934) of the point cloud. A filtration of the resulting simplicial complex is typically created by measuring the spatial scale of the simplices, similar to the Dowker filtration as described above.^5^

For a multispecies point cloud $P = \cup _{i = 0}^m P_i$, $P_i \cap P_j = \emptyset$ for $i \ne j$, we use a similar construction to capture the spatial patterns of different $P_i$. However, rather than choosing a subset of landmarks *L* from *P*, we use one of the point species as landmarks, i.e., $L = P_0$. Motivated by the close relationship of the witness complex and the Delaunay triangulation (de Silva and Carlsson 2004), we create the Delaunay triangulation (Delaunay 1934) $D_0$ on the landmark set, i.e., for 2D point cloud data we create the triangulation of the 2D convex hull of $P_0$. We include all simplices from the Delaunay triangulation and their faces in our simplicial complex, i.e., for 2D data we include all triangles, their edges, and their vertices as the 2-, 1- and 0-simplices of the simplicial complex. The remaining point species $P_i$ for $i = 1,...,m$ in *P* are then used as witnesses for the simplices in the Delaunay triangulation:

#### Definition 5

($P_i$-witness point) Let $p \in P_i$, $L \subset P$ be a landmark set, and *d* a distance function on *P*. We say that *p* is a $P_i$-*witness* for the *n*-simplex $\sigma = (l_0,\dots ,l_n)$ if $d(p,l_i) \le d(p,\hat{l})$ for all $\hat{l} \in L{\setminus }\{l_0,\dots ,l_n\}$ and $i = 0,...,n$.

We now create species-dependent filtrations $W^\bullet _{0,i}$ on the landmark set $P_0$ using witness points from $P_i$:

#### Definition 6

(Multispecies witness filtration $W^\bullet _{0, i}$) Let $P = \cup _{i = 0}^m P_i$ denote a collection of different point clouds, and let $D_0$ be the Delaunay triangulation of $P_0$. The *multispecies witness filtration* is a sequence of nested simplicial complexes $W^\bullet _{0, i}$ on $P_0$ with respect to witness points in $P_i$ where $W^\mu _{0,i}$ has $P_0$ as its potential vertex set and includes the *n*-simplex $\sigma = (p_0,\dots ,p_n) \in D_0$ and all its faces, if $\tilde{\mu }^\sigma \le \mu$ with $\tilde{\mu }^\sigma = \frac{\mu _{\text {max}}-\mu ^\sigma }{\mu _{\text {max}}}$, where $\mu ^\sigma$ is the number of $P_i$-witnesses of $\sigma$ and $\mu _{\text {max}}$ is the maximal number of $P_i$-witnesses for a simplex in $D_0$.

**Fig. 7:**
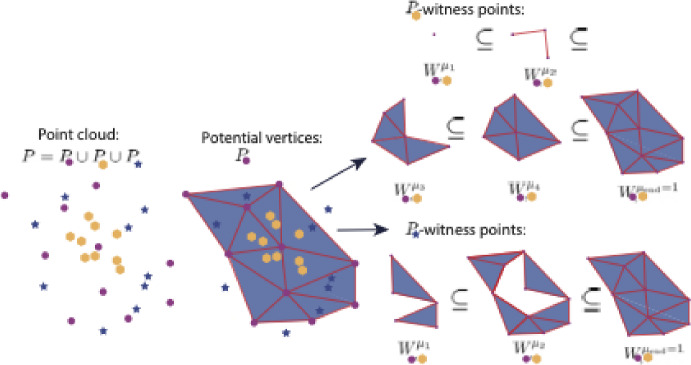
Example multispecies witness filtration. Given a point cloud $P = P_0 \cup P_1 \cup P_2$, we illustrate two multispecies witness filtrations on the Delaunay triangulation on $P_0$ using witness points from $P_1$ (depicted as yellow hexagons) and witness points from $P_2$ (depicted as blue stars). Different witness points give rise to different filtrations of the same simplicial complex (Color figure online)

We illustrate the multispecies witness filtration in an example point cloud in Fig. 7. Note that for the filtration to be well defined, it is necessary to assign filtration values such that all faces of newly added simplices in a particular filtration step are either already present in the filtration or are added in the same filtration step. It is not sufficient to compute only the filtration values of the top-dimensional simplices as their faces can have a higher number of witnesses (see Fig. 7 top row for an example).

To compare the effect of the different types of witnesses on the filtration, we first compute the dimension-0 and dimension-1 persistence diagrams of the multispecies witness filtrations, denoted $\text {pd}_0(W^\bullet _{0,i})$ and $\text {pd}_1(W^\bullet _{0,i})$, for $i=1, \dots , m$, and we compute pairwise distance vectors among the different persistence diagrams. We focus on distances between persistence diagrams. The entries of our distance vectors are given by the pairwise bottleneck distances $d_B$ among $\text {pd}_0(W^\bullet _{0,i})$, the pairwise bottleneck distances $d_B$ among $\text {pd}_1(W^\bullet _{0,i})$, the pairwise 1-Wasserstein distances $d_W$ among $\text {pd}_0(W^\bullet _{0,i})$, and the pairwise 1-Wasserstein distances $d_W$ among $\text {pd}_1(W^\bullet _{0,i})$ for $i=1, \dots , m$. Given a point cloud $P = \bigcup _{i=0}^m P_i$ with $m+1$ species, this results in distance vectors with $2 \times 2 \times {m \atopwithdelims ()2} = 2\,m(m-1)$ entries. We note that this choice of distance vector sidesteps the additional steps (and parameter choices) of constructing other vectorizations such as persistence images or persistence landscapes. Note that using different witness points leads to differences in the filtrations of the Delaunay triangulation of $P_0$ (see Fig. 7). Taking distances directly between the filtrations allows us to capture relative changes in spatial relations that are more relevant to our interpretation than the topological features of the filtrations themselves.

## Results

We demonstrate the utility of relational PH in predicting the macrophage phenotype (Problem 1) and in classifying the qualitative behavior of different parameter regimes of the ABM (Problem 2). For the first task, we find that using Dowker PH features improves the performance of a classifier in comparison to using both non-relational topological and non-topological features. In particular, we find that Dowker PH between tumor cells and blood vessels is the best predictor for the dominant macrophage phenotype. For the second task, we perform classification using the multispecies witness filtration features and recover the previous subjective classification of Fig. 3.

### Dowker Persistent Homology Predicts Dominant Macrophage Phenotype

#### Prediction Pipeline

**Fig. 8:**
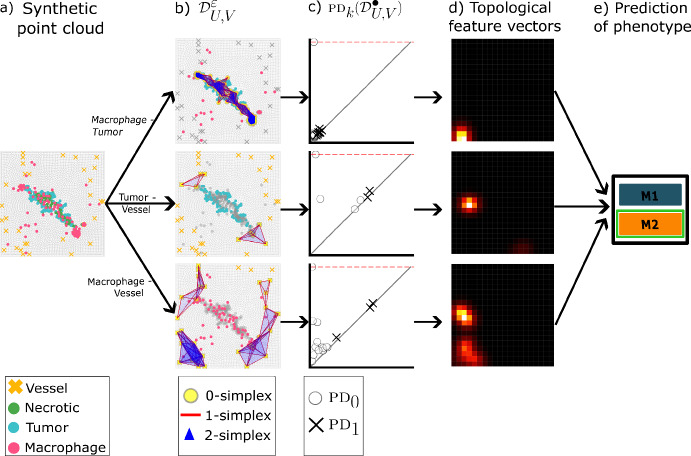
Pipeline for macrophage phenotype prediction using Dowker PH. **a** A point cloud representing a synthetic tumor microenvironment generated by an ABM. **b** Dowker complexes built on different pairs of cells at fixed proximity parameters. **c** Dowker persistence diagrams $\text {pd}_k(\mathcal {D}_{U,V}^{\bullet })$ for $k = 0, 1$. **d** Vectorization of (dimension-0) Dowker persistence diagrams via persistence images. **e** An SVM classifier takes a concatenation of flattened persistence image as input and predicts the dominant macrophage phenotype of the synthetic tumor microenvironment

We classify a synthetic tumor microenvironment as either anti-tumor ($M_1$) macrophage dominant or pro-tumor ($M_2$) macrophage dominant based on the spatial distributions of blood vessels, tumor cells, and macrophages. Since the $M_1$ and $M_2$ macrophages exhibit significantly different dynamics in the tumor microenvironment (see Sect. 2.1), we hypothesize that the relations of spatial distributions among the three cell types are good predictors of the dominant macrophage phenotype. Our input data is a point cloud $P = P_V \cup P_T \cup P_M$ that represents the locations of the three cell types. Note that the input data is blind to the phenotype of individual macrophages.

Given a point cloud *P*, if 50% or more macrophages are $M_1$ macrophages, then we label the point cloud as $M_1$ dominant. Otherwise, we label the point cloud as $M_2$ dominant. A total of 731 images are labeled 0 ($M_1$ dominant), and 241 images are labeled 1 ($M_2$ dominant).

For each *P*, we use Dowker PH to capture relations between pairs of constituents of the tumor microenvironment (see Fig. 8a,b). Note that only the spatial information of macrophages, and not the macrophage phenotype, is used to create the topological descriptors. We consider the following three pairs of cell types: macrophages and tumor cells, tumor cells and blood vessels, and macrophages and blood vessels (see Fig. 8b). For each pair, we compute the dimension-0 and dimension-1 Dowker persistence diagrams. Since the Dowker persistence diagram is agnostic to the choice of the vertex set (Theorem 2), the vertex set was chosen to be the cell type with a smaller number of points for faster computation (see Fig. 8c). Each point cloud thus results in six Dowker persistence diagrams: $\text {pd}_0(\mathcal {D}_{M,V}^{\bullet })$, $\text {pd}_1(\mathcal {D}_{M,V}^{\bullet })$, $\text {pd}_0(\mathcal {D}_{T,V}^{\bullet })$, $\text {pd}_1(\mathcal {D}_{T,V}^{\bullet })$, $\text {pd}_0(\mathcal {D}_{M,T}^{\bullet })$, $\text {pd}_1(\mathcal {D}_{M,T}^{\bullet })$.

Each Dowker persistence diagram is vectorized via persistence images to an array of size 20 $\times$ 20^6^ (see Fig. 8d) and flattened into vectors of size 400. We concatenate the resulting vectors and train a Support Vector Machine (SVM) for the image classification task. (see Fig. 8e).

We also train SVMs on non-relational topological features obtained from four Vietoris-Rips persistence diagrams: $\text {pd}_0(\mathcal{V}\mathcal{R}^{\bullet }_{T})$, $\text {pd}_1(\mathcal{V}\mathcal{R}^{\bullet }_{T})$, $\text {pd}_0(\mathcal{V}\mathcal{R}^{\bullet }_{M})$, $\text {pd}_1(\mathcal{V}\mathcal{R}^{\bullet }_{M})$. We further train an SVM on non-topological features such as the count of each cell type and the average distance of each cell type to the nearest blood vessels (see data description in Sect. 2.1).

For each SVM classifier, we optimize the hyperparameters via stratified 5-fold cross-validation, employing the synthetic minority oversampling technique (SMOTE) (Chawla et al 2002) in each fold to address the class imbalance. We train an SVM on 10 different random splits of train and test data and report the 10 classification accuracies on the test data.

#### Dowker Persistence Diagrams Capture Shared Topological Features

**Fig. 9:**
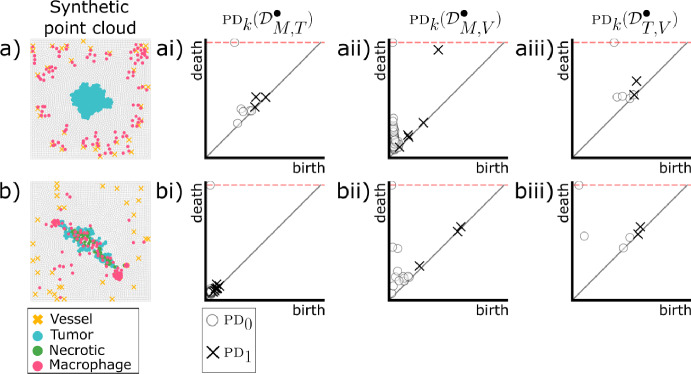
Dowker persistence diagrams capture spatial relations between cell types. **a** A synthetic tumor microenvironment in which macrophages and blood vessels surround a compact tumor. The six Dowker persistence diagrams are shown in **ai**, **aii**, **aiii**. **b** A synthetic tumor microenvironment where the cancer cells and macrophages occupy different spaces from the blood vessels. The cancer cells and macrophages are in close proximity to blood vessels in two regions – the top left and bottom right corners of the tumor mass. The six Dowker persistence diagrams are shown in **bi**, **bii**, **biii**. **ai** The large birth parameters of points in $\text {pd}_0(\mathcal {D}_{M,T}^\bullet )$ indicate that macrophages and tumor cells are far from one another. **aii** The small birth parameters of points in $\text {pd}_0(\mathcal {D}_{M,V}^{\bullet })$ indicate that macrophages and blood vessels are colocalized. The single cross far from the diagonal in $\text {pd}_1(\mathcal {D}_{M,V}^{\bullet })$ indicates that macrophages and blood vessels share a common loop. **aiii** Both Dowker persistence diagrams are similar to the diagrams in panel **ai** because the relationship between tumor cells and blood vessels is similar to the relationship between macrophages and tumor cells. **bi** The small birth parameters of $\text {pd}_0(\mathcal {D}_{M,T}^{\bullet })$ indicate that macrophages and tumor cells occupy similar regions. **bii** The spread of birth parameters for points in $\text {pd}_0(\mathcal {D}_{M,V}^{\bullet })$ indicates the variance in the extent to which macrophages and vessels occupy similar spaces. **biii** The two points in $\text {pd}_0(\mathcal {D}_{T,V}^{\bullet })$ far from the diagonal indicate that there are two regions (the top left and bottom right corners of the tumor mass) where the tumor cells and the blood vessels are close to each other

Before we discuss classification accuracy, we present example point clouds and interpretation of Dowker persistence diagrams (see Fig. 9).

Recall that $\text {pd}_0(\mathcal {D}_{U,V}^{\bullet })$ summarizes the birth and death of connected components of Dowker complexes as one varies the distances between $P_U$ and $P_V$. One can thus consider a dimension-0 Dowker persistence diagram as summarizing shared connected components between two point clouds. There are multiple ways in which a shared connected component arises - $P_U$ and $P_V$ might occupy a similar region, or $P_U$ and $P_V$ may occupy different regions but have close contact. In such cases, the shared features will be represented by points in $\text {pd}_0(\mathcal {D}_{U,V}^{\bullet })$ with small birth parameters.

For example, consider the relationship between macrophages and tumor cells in Fig. 9a and Fig. 9b. In Fig. 9a, the macrophages are distant from the tumor cells, so the points in $\text {pd}_0(\mathcal {D}_{M,T}^{\bullet })$ have large birth times (see Fig. 9ai). On the other hand, in Fig. 9b, the macrophages and tumor cells occupy similar spaces, so the points in $\text {pd}_0(\mathcal {D}_{M,T}^{\bullet })$ have small birth times (see Fig. 9bi).

Consider the relationship between tumor cells and blood vessels in Fig. 9b. The tumor cells and blood vessels mostly occupy different spaces. However, the tumor cells and blood vessels are in close proximity in two regions, one on the top left corner and another on the bottom right corner of the tumor mass. The fact that there are two “contact points” between the tumor and blood vessels is reflected by two points in $\text {pd}_0(\mathcal {D}_{T,V}^{\bullet })$ that are far from the diagonal (Fig. 9biii). In Fig. 9a, the macrophages and blood vessels occupy very similar regions. Such colocalization between macrophages and blood vessels is reflected by the abundance of points in $\text {pd}_0(\mathcal {D}_{M,V}^{\bullet })$ with small birth times (see Fig. 9aii).

A dimension-1 Dowker persistence diagram summarizes the evolution of cycles of Dowker complexes as one varies the distances between *U* and *V*. We interpret points in $\text {pd}_1(\mathcal {D}_{U,V}^{\bullet })$ that are far from the diagonal line as representing shared loops between two point clouds. For example, the macrophages and blood vessels in Fig. 9a share a loop structure, and such shared loop is reflected by a point in $\text {pd}_1(\mathcal {D}_{M,V}^{\bullet })$ that is far from the diagonal (Fig. 9aii). We caution the reader that $\text {pd}_1(\mathcal {D}_{U,V}^{\bullet })$ may contain points far from the diagonal line even if $P_U$ and $P_V$ do not have shared cycles. Such a situation arises, for example, when $P_U$ is sampled from a circle while $P_V$ is a dense, uniform sample of the background.

#### SVM on Dowker Features Predicts Dominant Macrophage Phenotype

**Fig. 10:**
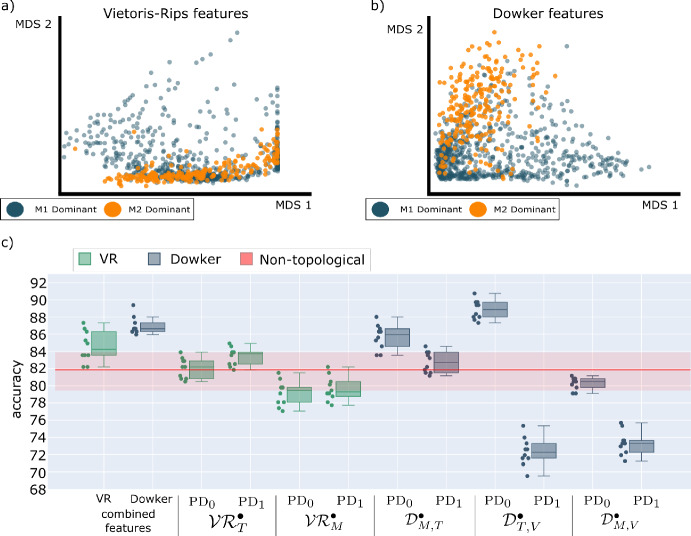
Dowker persistent homology features improve the prediction of dominant macrophage subtype. **a** MDS projection of Vietoris-Rips features. **b** MDS projection of Dowker features. The two classes have better separation when using Dowker features than the Vietoris-Rips features. **c** Classification accuracies of SVMs trained on Vietoris-Rips features (green), Dowker features (navy), and non-topological features (red). The box plot summarizes the accuracies from 10 different splits of train and test data. The red box shows the minimum (lower bounding line), median (middle line), and maximum (upper bounding line) accuracy values for SVM trained on non-topological feature vectors. The first two box plots show the accuracies of two SVMs, one trained on concatenated Vietoris-Rips features and another trained on concatenated Dowker features. SVM trained on Dowker features has higher accuracy than SVM trained on Vietoris-Rips features. The remaining box plots show the accuracies of SVMs trained on individual Vietoris-Rips or Dowker features. SVM trained on $\text {pd}_0(\mathcal {D}^\bullet _{T, V})$ has the highest accuracy among all SVM trained on the Dowker features (Color figure online)

We first visually inspected whether Dowker persistence diagrams can distinguish $M_1$ and $M_2$ dominant tumor microenvironments. Recall that we computed six Dowker persistence diagrams, which resulted in six 400-dimensional vectors. We refer to the six vectors as Dowker features. We concatenated the six vectors into a 2400-dimensional vector, and we refer to the resulting vector as a concatenated Dowker feature vector. A two-dimensional visualization via Multidimensional Scaling (MDS) (Kruskal 1964) shows decent separation of classes (see Fig. 10b). For comparison, we computed four Vietoris-Rips persistence diagrams from tumor cells and macrophages and vectorized them. We refer to the four vectors as Vietoris-Rips features. We refer to the concatenated vectors as a concatenated Vietoris-Rips feature vector. Visualization of MDS (Fig. 10a, b) indicates that the concatenated Dowker feature vectors may be better predictors of the dominant macrophage phenotype.

We train two SVM classifiers, one that takes the concatenated Dowker feature vector as input and another that takes the concatenated Vietoris-Rips feature vector as input. The SVM trained on concatenated Dowker features has higher accuracy (median accuracy $86.6\%$) than the SVM trained on the concatenated Vietoris-Rips features (median accuracy $84.2\%$). Furthermore, the lower quartile of accuracy from the concatenated Dowker features is roughly equal to the upper quartile of accuracy from the concatenated Vietoris-Rips features ($\sim 86\%$) (see Fig. 10c). Both models outperform an SVM trained on non-topological features such as the number of cells per cell type and average distances of cell types to the nearest blood vessels (see Fig. 10c).

Next, we investigate which cell types were most informative in predicting the dominant macrophage of the synthetic tumor microenvironment. To this end, we train ten additional SVM classifiers on the Dowker and Vietoris-Rips features without any concatenation. We train four classifiers on the four Vietoris-Rips features and six classifiers on the six Dowker features. Among the classifiers trained on Vietoris-Rips features, the model trained on $\text {pd}_1(\mathcal{V}\mathcal{R}^{\bullet }_{T})$ has the highest median accuracy ($83.7 \%$). One possible explanation is that $M_2$ macrophages assist metastasis of tumor cells by guiding them away from the tumor mass towards the blood vessels. During this process, the tumor cells may create many small loops as they navigate away from the tumor mass, creating many non-trivial points in $\text {pd}_1(\mathcal{V}\mathcal{R}^{\bullet }_{T})$. The persistence diagram $\text {pd}_1(\mathcal{V}\mathcal{R}^{\bullet }_{T})$ may then reflect the extent to which $M_2$ macrophages assist the spread of cancer cells.

Among the classifiers trained on Dowker features, the model trained on $\text {pd}_0(\mathcal {D}_{T,V}^{\bullet })$ has the highest accuracy (median accuracy $88.9\%$), followed by the model trained on $\text {pd}_0(\mathcal {D}_{T,M}^{\bullet })$ ($86.0 \%$). It is perhaps surprising that the best predictor of the dominant macrophage phenotype uses the relations between tumor cells and blood vessels and not macrophages. One possible explanation for the improved performance of models using $\text {pd}_0(\mathcal {D}_{T,V}^{\bullet })$ in our application is that $\text {pd}_0(\mathcal {D}_{T,V}^{\bullet })$ captures colocalization between tumor cells and blood vessels, which can represent the extent to which $M_2$ macrophages have assisted the tumor cells to navigate towards blood vessels for metastasis.

Note that dimension-1 Dowker features involving blood vessels are not particularly good predictors of the dominant macrophage phenotype (see Fig. 10c). The poor performance may be due to the lack of common loops between blood vessels and tumor cells and between the blood vessels and the macrophages.

The analysis at individual time points shows that the performance of the classifiers depends on the time point. In particular, there are time points at which the relational features are more valuable than the Vietoris-Rips features in predicting the dominant macrophage subtype (see SI Fig.4).

### Multispecies Witness Features Identify Qualitative Model Behaviors

**Fig. 11:**
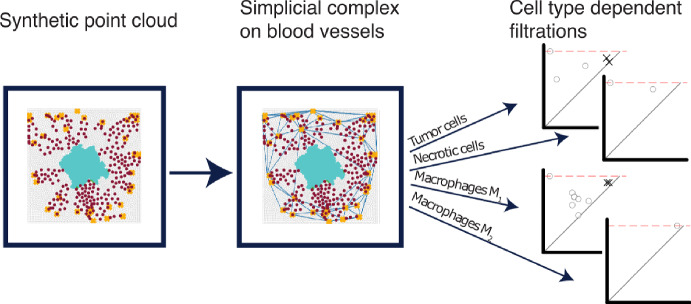
Multispecies witness PH on synthetic data from ABM. The point cloud given by the synthetic data $P = P_{\text {V}} \cup P_{\text {T}} \cup P_{\text {N}} \cup P_{M_1} \cup P_{M_2}$ consists of blood vessels $P_V$, tumor cells $P_{\text {T}}$, necrotic cells $P_{\text {N}}$, anti-tumor macrophages $P_{M_1}$ and pro-tumor macrophages $P_{M_2}$. We construct a Delaunay triangulation on the blood vessels $P_{\text {V}}$ and build cell type dependent filtrations $W_{V,i}^\bullet$ of the Delaunay triangulation where $i \in \{\text {T},\text {N},M_1,M_2 \}$. We obtain one persistence diagram for each cell type-specific filtration

To study the different qualitative behaviors of the ABM, we focused on differences between the spatial distributions of the different cell types and applied the multispecies witness PH. We illustrate how we applied multispecies witness PH to the output of our ABM in Fig. 11.

**Fig. 12:**
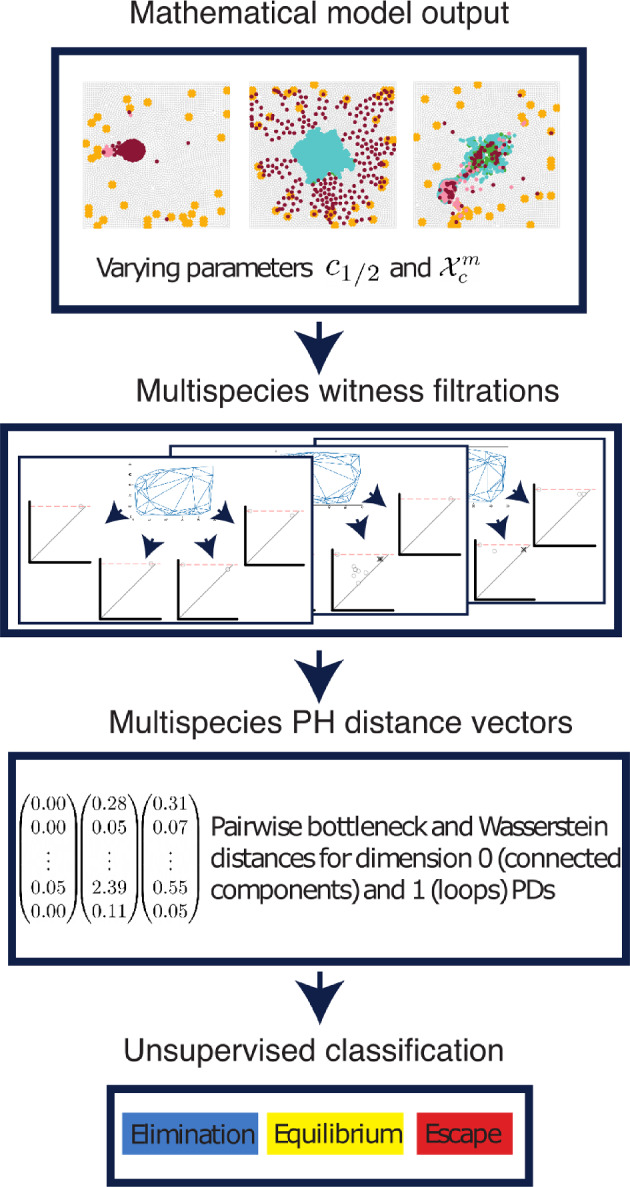
Multispecies witness PH pipeline. We use the point cloud generated by an ABM as input into our multispecies witness filtrations. We compute persistence diagrams for the multispecies witness filtrations, thereby obtaining topological descriptors of the spatial heterogeneity in the input images. We use the persistence diagrams to compute multispecies PH distance vectors. The entries of these vectors correspond to the pairwise bottleneck and 1-Wasserstein distances between the dimension-0 and dimension-1 persistence diagrams of the cell type-specific filtrations. We use the multispecies PH distance vectors as input into unsupervised classification to identify different qualitative behaviors of the ABM

Our point cloud data $P = P_{\text {V}} \cup P_{\text {T}} \cup P_{\text {N}} \cup P_{M_1} \cup P_{M_2}$ consists of blood vessels $P_V$, tumor cells $P_{\text {T}}$, necrotic cells $P_{\text {N}}$, anti-tumor macrophages $P_{M_1}$ and pro-tumor macrophages $P_{M_2}$. We chose to fix $P_0 = P_V$ and considered two different versions for the witness filtrations: first, we did not distinguish macrophage phenotype, i.e., all macrophages are assumed to be identical and $P_{\text {M}} = P_{M_1} \cup P_{M_2}$. We obtained three different witness filtrations using tumor cells, necrotic cells, and macrophages as witness points. In the second case, we distinguished $M_1$ and $M_2$ macrophage subtypes and constructed four witness filtrations using tumor cells, necrotic cells, $M_1$ macrophages, and $M_2$ and macrophages as witness points. From the persistence diagrams, we computed multispecies PH distance vectors (see Sect. 4.2) to compare the effect of the different types of witnesses on the filtration. The entries of our distance vectors are listed in SI Table 1. We choose a combination of entries corresponding to the 1-Wasserstein distance and the bottleneck distance. This allows us to indirectly include the spatial relations of the witness cell types and the fixed blood vessel point cloud (via the sensitivity of the 1-Wasserstein distance to the number of features in a persistence diagram) while also capturing particularly large changes in individual topological features across the different witness cell type filtrations (via the bottleneck distance). The pairwise distances each contributed 3 entries when all macrophages are considered to be the same cell type and 6 entries when distinguishing between $M_1$ and $M_2$ macrophages for each topological dimension considered. In this way, we converted each point cloud *P* into a 12- (version 1) and a 24-dimensional (version 2) distance vector, respectively (for a summary, see SI Table 1). We used these distance vectors as input into *k*-means clustering (specifically, we apply the *k*-means version implemented in sklearn.cluster which uses Euclidean distance in the clustering algorithm). We summarize the full multispecies witness PH pipeline in Fig. 12. We compared our results to clustering performed on simple (non-topological) descriptor vectors (see data description in Sect. 2.1 for description of simple vectors and see SI Fig. 1 for results).

#### Multispecies Witness Persistence Classification Disregarding Macrophage Subtype

**Fig. 13:**
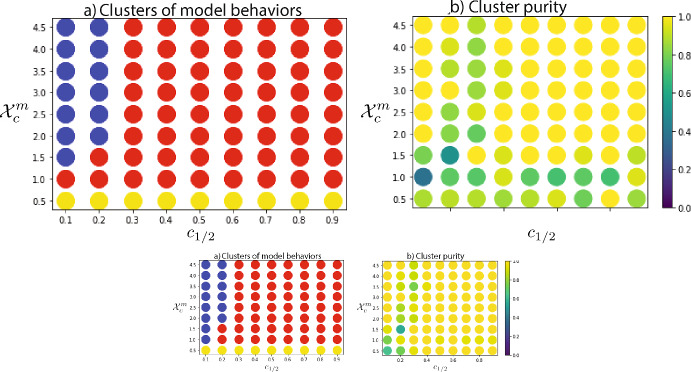
Classification of multispecies PH distance without distinguishing between macrophage subtypes. **a** Classification results. **b** Cluster purity scores. For each parameter combination $\mathcal {X}^m_c$ and $c_{1/2}$ of the ABM, we include 20 independent simulations in our analysis. The colors red, blue, and yellow represent the cluster to which the majority of simulations are attributed by the *k*-means algorithm for $k=3$. The purity score is computed by taking the ratio between the number of simulations attributed to the majority clusters and the total number of 20 simulations (Color figure online)

We recovered the three qualitatively different behaviors of the ABM using the unsupervised multispecies witness PH pipeline without including knowledge about macrophage subtypes. We applied *k*-means classification for $k = 3$. Figure 13 shows which of the three clusters is dominant amongst the 20 simulations for each parameter combination of $\mathcal {X}^m_c$ and $c_{1/2}$ that we consider. The results are consistent with the subjective classification of the qualitative behaviors of the model shown in Fig. 3, i.e., we recovered parameter regimes dominated by tumor elimination, tumor macrophage equilibrium, and escape of the tumor, with the exception of simulations in regimes at the boundaries between the three behaviors. We investigated the consistency of the cluster assignment, which we refer to as *cluster purity* by dividing the number of simulations attributed to the majority cluster by the total number of simulations for the parameter combination. We found that cluster assignment is less consistent in simulations of the ABM that lie in boundary regions between different qualitative behaviors than in parameter regimes far away from boundaries (see Fig. 13). Our results clearly surpass clustering obtained using simple descriptor vectors of the data (see SI Fig. 1), including information such as the number of cells per cell type and average distances of cell types to the nearest blood vessels with respect to cluster consistency with the subjective clusters shown in Fig. 3.

#### Multispecies Witness Persistence Classification Including Macrophage Subtypes

**Fig. 14:**
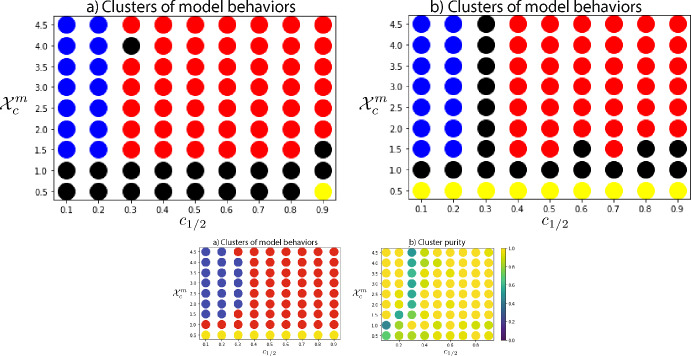
Classification of multispecies PH distance vectors distinguishing between macrophage subtypes $M_1$ and $M_2$. **a** Classification results. We highlight parameter combinations for which the classification changed due to the inclusion of macrophage subtype by using a square symbol as opposed to a circle. **b** Cluster purity scores. For each parameter combination $\mathcal {X}^m_c$ and $c_{1/2}$ of the ABM, we include 20 independent simulations in our analysis. The colors red, blue, and yellow represent the cluster to which the majority of simulations are attributed by the *k*-means algorithm for $k=3$. The purity score is computed by taking the ratio between the number of simulations attributed to the majority clusters and the total number of 20 simulations (Color figure online)

We also recovered the three qualitatively different behaviors of the ABM when information about macrophage subtypes $M_1$ and $M_2$ is included in the construction of our multispecies PH distance vectors. We show our results in Fig. 14. Comparison of the results in Fig. 13 and Fig. 14 shows that the inclusion of the additional information about macrophage subtype alters the prediction of the qualitative behaviors for only one parameter combination (see combination highlighted by square symbol in Fig. 14), $\mathcal {X}^m_c = 1$ and $c_{1/2} = 0.1$, which is located at the phase transition between elimination and escape. We also computed the purity of clusters for each parameter combination by dividing the number of simulations attributed to the majority cluster by the total number of simulations for the parameter combination. We find that clusters assigned to parameter combinations located at the phase transitions between different parameter regimes are less consistent than those far away from boundaries. Again, our results surpass clustering obtained using simple descriptor vectors of the data, including information such as the number of cells per cell type and average distances of cell types to the nearest blood vessels (see SI Fig. 1 for results).

#### Multispecies Witness Persistence Classification Determines Phase Transitions as Separate Cluster

**Fig. 15:**
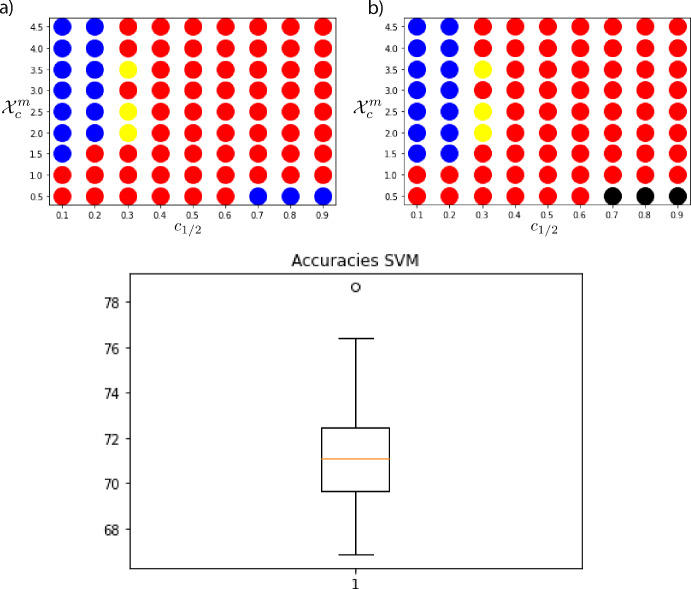
Classification of multispecies PH distance vectors for $k=4$ in *k*-means clustering. **a** Classification results without knowledge of macrophage subtypes. **b** Classification results when distinguishing between macrophage subtypes $M_1$ and $M_2$. The colors red, blue, yellow, and black represent the cluster to which the majority of simulations are attributed by the *k*-means algorithm for $k=4$ (Color figure online)

Multispecies PH distance vectors further stratified the parameter space of the ABM not only into the three qualitatively different behaviors but also into the regions of phase transitions. When applying *k*-means classification for $k = 4$, the phase transitions between qualitative behaviors were identified as a separate cluster when including macrophage subtypes $M_1$ and $M_2$ in the analysis (see Fig. 15 b). Interestingly, when ignoring macrophage subtypes (see Fig. 15 a), this effect was less prominent. These results could not be obtained when using *k*-means classification for $k = 4$ on simple descriptor vectors of the data including information such as the number of cells per cell type and average distances of cell types to the nearest blood vessels (see SI Fig. 1).

#### Multispecies Witness Persistence Classification is Robust to Mislabeling of Cell Types

The multispecies witness PH pipeline is robust to noise introduced through relabeling. For each point cloud generated by the ABM, we relabeled up to $50\%$ of the necrotic cells, $M_1$, and $M_2$ macrophages. Relabeled cells were randomly attributed the label of one of the other two cell types. For example, a necrotic cell had a $50\%$ chance of being relabelled as a $M_1$ or $M_2$ macrophage. We focused on these three cell types because their numbers are of comparable magnitude in the ABM output, e.g., relabeling tumor cells or vessels would lead to the addition of a disproportionately high or low number of the other three cell types to the simulation output.

**Fig. 16:**
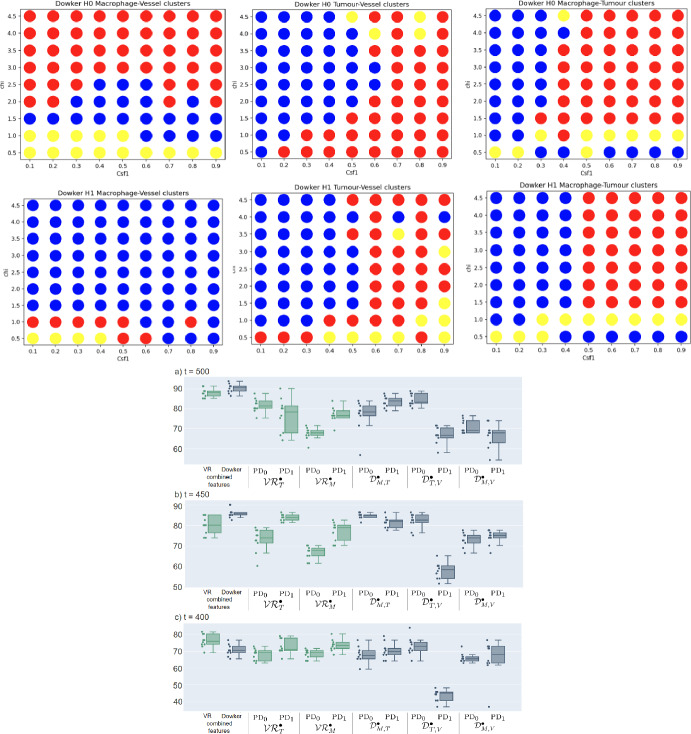
Classification of multispecies PH distance vectors after relabeling $50\%$ of the data. **a** Classification results. We highlight parameter combinations for which the relabelling changes the classification (compared to multispecies PH distance vectors distinguishing $M_1$ versus $M_2$ macrophages) using a square symbol as opposed to a circle. **b** Cluster purity scores. For each parameter combination $\mathcal {X}^m_c$ and $c_{1/2}$ of the ABM, we include 20 independent simulations in our analysis. The colors red, blue, and yellow represent the cluster to which the majority of simulations are attributed by the *k*-means algorithm for $k=3$. The purity score is computed by taking the ratio between the number of simulations attributed to the majority clusters and the total number of 20 simulations

## Discussion

With the advancement of data collection techniques, there is a growing need for analysis tools that extract relational information from spatial multispecies data. We presented two novel topological approaches to study structural relations: Dowker PH and multispecies witness PH. Dowker PH produces interpretable persistence images, but its application is limited to pairwise relations. Multispecies witness PH, on the other hand, produces features that are more difficult to interpret, but it captures relations among three or more species. The two topological methods were incorporated into two example pipelines that can be adjusted with different choices of vectorizations. We tested the utility of relational topological features in understanding macrophage and tumor behavior in point cloud simulations of the tumor microenvironment. Our results show that topological relations provide biological insight beyond that contributed by non-relational topological features and non-topological features. Furthermore, our study demonstrates that Dowker PH and multispecies witness PH effectively encode topological relations.

This study contributes novel tools for capturing topological spatial relations that are missed in standard methods. A comparison of topological quantifications of relations to various spatial statistics (Wilson et al 2021), including the recently introduced weighted pair-correlation function (Bull and Byrne 2023), is postponed for future research. We believe that the topological methods, when combined with the computation of cycle representatives, may provide extra insight by identifying the local regions at which relational topological features occur in a point cloud.

Other viable topological methods include multiparameter persistence (Vipond et al 2021) and the chromatic alpha complex (di Montesano et al 2022). Multiparameter persistence creates multifiltrations of a simplicial complex using properties such as distances and density; one could potentially use within-species distance and cross-species distance to create such multifiltrations. However, there are many practical limitations to utilizing multiparameter persistence for multispecies data, such as computability and interpretability. The chromatic alpha complex (di Montesano et al 2022) creates a filtration on a multispecies version of the Delaunay triangulation. While the chromatic alpha complex is a viable and interesting approach for studying multispecies data, it will be computationally more expensive than the multispecies witness filtration since the chromatic alpha complex builds a Delaunay complex on the full point cloud, whereas the multispecies witness filtration builds a Delaunay triangulation on one of the point species. Furthermore, the chromatic alpha complex involves an additional convex optimization step for computing the radius function, whereas the filtrations in the multispecies witness filtration can be read directly from the distance matrix. We believe that the Dowker PH, the multispecies witness filtration, and the chromatic alpha complex characterize different types of spatial patterns, and a comparison of these approaches will be a topic of future study.

One of the limitations of the current work is that Dowker PH can be sensitive to outliers. For example, if $P_1$ and $P_2$ are point clouds that are excluded from one another, a single outlier point of $P_1$ that lives in the neighborhood of $P_2$ will create a shared feature that is encoded by the dimension-0 Dowker persistence diagram. An enhancement of Dowker PH for robustness against outliers, possibly through subsampling (Stolz 2023; Chazal et al 2015) and multiparameter persistence, is postponed for future work.

A further study could investigate the impact of choices in the pipelines, including the choice of the topological methods and the vectorizations. Here, Dowker PH, with its focus on shared topological features, captured the spatial relations underlying changes in the macrophage subtype, while multispecies witness PH successfully highlighted relative differences in spatial distributions across the different cell types. Motivated by the underlying biological problems, we made different choices of vectorizations for the two biological problems. The first choice focused on shared features (persistence images with Dowker PH) and the second method highlighted the differences in spatial patterns (distance vectors with multispecies witness PH). It is important to adapt these combinations to the context of the application. For example, using Dowker PH to address the second biological question and using the multispecies witness PH to address the first biological question led to a decrease in performance (see SI Fig.2 and 3). Using multispecies witness PH for the second question decreased the median accuracy in predicting the dominant macrophage subtype from a median accuracy of 86% to an accuracy of 71% (compare Fig. 10 and SI Fig.2). As the macrophage subtypes are influenced by cellular interactions rather than the coarse differences in spatial distribution among different cell types, multispecies witness features were not as well-suited for this task as Dowker features. Conversely, Dowker features did not lead to a clustering of the three qualitative regimes that resemble the expert annotations (compare Fig. 13 and SI Fig.3. See Fig. 3 for expert annotations). We hypothesize that this difference in performance reflects that the three qualitative regimes in the ABM are more dependent on global differences in spatial distributions among different cell types, rather than shared topological features as captured by the Dowker complex.

A study of the choices in the construction of multispecies witness PH is left for future work. While the multispecies witness PH was based on the lazy witness complex, one could extend the construction to “non-lazy” witness complexes. When applying the multispecies witness PH to simulated tumor microenvironments, we chose the blood vessels as landmarks. Investigation into the influence of the landmark cell type, along with the possibility of using randomly selected points in the domain as landmarks, are subjects of future work.

Recent developments in imaging techniques (Goltsev et al 2018; Giesen et al 2014) and cell identification techniques (Pratapa et al 2021; Yao et al 2019) produce multispecies immunohistochemistry images with detailed information about the locations of various constituents of a tissue microenvironment. In tumor tissue, these constituents may include tumor cells, T-cells, B-cells, stroma, blood vessels, and more. Relational PH can potentially be applied to such multiplex images to automatically extract interpretable quantifications of relations among tumor constituents. Furthermore, the relational topological features can more broadly be applied to many other data sets that carry information on spatial locations of multiple systems. In the future, we envisage the integration of our methods with machine learning tools such as graph neural networks, deep learning, and random forests to achieve increased performance on such relational data and achieve novel insights.

## Supplementary Information

Below is the link to the electronic supplementary material.

Supplementary file 1 (pdf 1853 KB)

## Data Availability

The data is available in the accompanying materials of Bull and Byrne (2023). All code is available at https://github.com/irishryoon/multiplex_relations. The Dowker PH was computed in Julia using https://github.com/irishryoon/Dowker_persistence. We implemented the multispecies witness PH in Python using the gudhi library (Maria et al 2014) to compute persistence diagrams, as well as bottleneck and Wasserstein distances.

## Footnotes

1 Note that our construction differs from the lazy witness filtration where the simplices in $P_0$ and their filtration values are determined by their proximity to witnesses. Here, we use the number of witnesses to determine the filtration values on a Delaunay triangulation of $P_0$.

2 These come from 2 sets of 10 realizations in which the threshold value of TGF-$\beta$ required to change macrophage phenotype was varied (either 0.05 or 0.5). Varying this parameter had no qualitative effect on the simulations, and hence the parameter regimes have here been combined.

3 Note that the Wasserstein distance can be defined for any type of norm, a typical choice is $L_\texttt{p}$ for $\texttt{p} \in [1,\infty ]$.

4 Note that while the homology groups of the above constructions are isomorphic, their connectivity, as measured for example by *Q*-analysis (Atkin 1972), may differ.

5 The Dowker filtration can be viewed as a special case of the lazy witness filtration. In the general formulation of the lazy witness filtration (de Silva and Carlsson 2004), the distance to the $\nu$-th closest witness is added to the proximity filtration scale $\epsilon$. Given point clouds *P* and *Q*, a modified Dowker filtration in which all vertices have birth time 0 is a witness filtration with *P* as landmarks, *Q* as witnesses, and $\nu = 0$.

6 The classification accuracy is fairly robust to the size of the persistence image. Such robustness is known in the literature Adams et al (2017).
