# Supplementary material for: Relational Persistent Homology for Multispecies Data with Application to the Tumor Microenvironment
Source: Bull Math Biol. 2024 Sep 17;86(11):128. doi: 10.1007/s11538-024-01353-6 (PMC11408586; doi:10.1007/s11538-024-01353-6)
Supplement: Supplementary file 1 — (pdf 1853 KB) [file 11538_2024_1353_MOESM1_ESM.pdf]

Relational persistent homology for multispecies data with application to the tumor microenvironment

Bernadette J. Stolz[1,2†], Jagdeep Dhesi[2†], Joshua A. Bull[2], Heather A. Harrington[2,3], Helen M. Byrne[2,4] and Iris H.R. Yoon[2,5*]

[1]Laboratory for Topology and Neuroscience, EPFL, Station 8, Lausanne, 1015, Switzerland.
[2]Mathematical Institute, University of Oxford, Andrew Wiles Building, Woodstock Rd, Oxford, OX2 6GG, United Kingdom.
[3]Wellcome Centre for Human Genetics, University of Oxford, Roosevelt Dr, Headington, Headington, Oxford, OX3 7BN, United Kingdom.
[4]Ludwig Institute for Cancer Research, University of Oxford, Old Road Campus Research Build, Roosevelt Dr, Headington, Oxford, OX3 7DQ, United Kingdom.
[5]Department of Mathematics and Computer Science, Wesleyan University, 265 Church Street, Middletown, 06459, United States of America.

*Corresponding author(s). E-mail(s): hyoon@wesleyan.edu;
Contributing authors: bernadette.stolz-pretzer@epfl.ch; jaydhesi24@yahoo.co.uk; bull@maths.ox.ac.uk; harrington@maths.ox.ac.uk; helen.byrne@maths.ox.ac.uk;
†These authors contributed equally to this work.

Supplementary Information

1 Agent-based model

We consider tumor microenvironments generated by an ABM (Bonabeau, 2002), which simulates the behavior of a system by the decisions and interactions of the agents. Our model, described in (Bull and Byrne, 2023), simulates a growing tumor. The model explores how interactions between macrophages and the tumor microenvironment can generate interplay between varying macrophage phenotypes and the migration of tumor cells towards surrounding vasculature, a trait associated with tumor metastasis.

The model is a 2D, off-lattice, hybrid, force-based model, containing four different cell types (tumor cells, stromal cells, macrophages, and necrotic cells). Cell movement is determined by force-based interactions with neighbouring cells, together with interactions with five different chemical species described by partial differential equations (oxygen, CSF-1, TGF-β, CXCL12, and EGF). Blood vessels are represented as fixed points that are interpreted as cross-sections of vessels rising through the simulation plane.

A key part of the model is the phenotype label Ω associated with each macrophage. The macrophage phenotype varies continuously between 0 and 1. Macrophages with $\Omega \approx 0$ are anti-tumor 'M_1' macrophages which kill tumor cells on contact. On the other hand, 'M_2' macrophages with $\Omega \approx 1$ are pro-tumor and produce a chemokine, EGF, which increases tumor cell migration.

Availability of oxygen mediates the cell cycle of tumor and stromal cells, with lower oxygen availability causing reduced proliferation and, with sustained lack of oxygen, death. Dead cells are labelled as necrotic, and occupy space for a period of time. CSF-1 and CXCL12 are key chemokines for macrophages, and macrophages are attracted via chemotaxis towards increasing gradients of these chemicals. Crucially, M_1 macrophages are more strongly attracted towards CSF-1 (produced by tumor cells) while M_2 macrophages are more strongly attracted towards CXCL12 generated by perivascular fibroblasts (assumed to be co-located with blood vessels, and therefore not explicitly included as agents in the model). Macrophages enter the simulation through the vasculature with a phenotype $\Omega = 0$, and are attracted towards the tumor via the CSF-1 gradient. On reaching the tumor, they are exposed to TGF-β generated by tumor cells. Prolonged exposure to TGF-β causes macrophage phenotype to irreversibly increase, until it reaches a maximum of $\Omega = 1$. This reduces macrophage killing of tumor cells, and ultimately sensitizes them to the CXCL12 gradient produced from blood vessels, causing migration of M_2 macrophages back towards the vasculature. Since M_2 macrophages produce EGF, tumor cells can follow this gradient and may ultimately reach the vasculature (a trait associated with increased likelihood of tumor metastasis, which requires tumor cells to enter vasculature to migrate to other parts of the body).

We consider a parameter sweep in which two key parameters related to CSF-1 are varied: χ_c^m, the chemotactic sensitivity of macrophages to gradients of CSF-1, and $c_{1/2}$, the concentration of CSF-1 at which macrophage extravasation is half-maximal. All other parameters are held at constant values described in (Bull and Byrne, 2023). In Fig. 3a of the main-text we show subjective classification of different qualitative behaviours of the model resulting from different parameter regimes. These qualitative behaviours manifest in different spatial distributions of the different cell types. In particular, Bull and Byrne (2023) relate these to the three E's of cancer immunoediting (Dunn et al, 2004): low $c_{1/2}$ leads to tumor 'Elimination' as macrophages are highly recruited to the simulation and destroy the tumor. The exception to this is when χ_c^m is also low, generating 'Equilibrium' behaviour as macrophages are not sufficiently attracted to the tumor to destroy it. When χ_c^m and $c_{1/2}$ are both sufficiently high, M_1 macrophages are converted to M_2 macrophages faster than they can eliminate the tumor, causing tumor progression to the vasculature and thus immune 'Escape'.

2 Supplementary figures and table

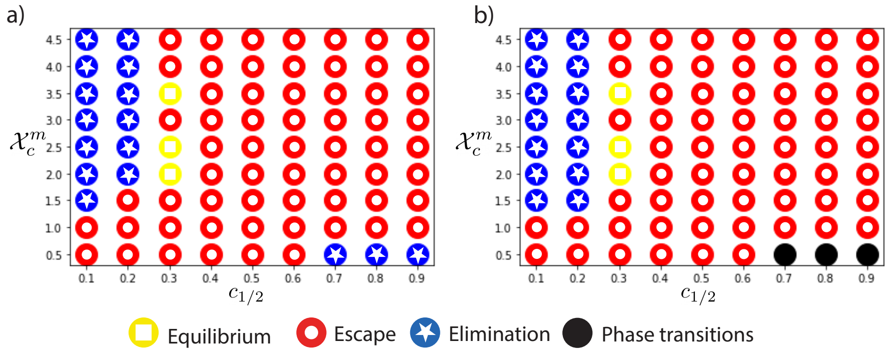

Fig. 1 Classification of simple feature vectors which do not incorporate spatial information of cell types. We apply k-means clustering to simple descriptor vectors for $k = 3$ (**a**) and $k = 4$ (**b**). We populate the simple description vectors with entries corresponding to the number of tumor cells, the number of macrophages, the number of necrotic cells, the average distance of tumor cells to the nearest blood vessel, the average distance of necrotic cells to the nearest blood vessel, and the average distance of macrophages to the nearest blood vessel

Table 1 Different versions of the multispecies witness PH and distance vectors. We populate the multispecies distance vectors with pairwise Bottleneck d_B and 1-Wasserstein distances d_W for persistence diagrams PD_i in dimensions $i = 0, 1$

Version	Witness filtrations considered	Distance vector entries
1	tumor cells, necrotic cells, and macrophages	$\begin{pmatrix} d_B(\text{PD}_i(W^{\bullet}_{V,T}), \text{PD}_i(W^{\bullet}_{V,N})) \\ d_B(\text{PD}_i(W^{\bullet}_{V,T}), \text{PD}_i(W^{\bullet}_{V,M})) \\ d_B(\text{PD}_i(W^{\bullet}_{V,N}), \text{PD}_i(W^{\bullet}_{V,M})) \\ d_W(\text{PD}_i(W^{\bullet}_{V,T}), \text{PD}_i(W^{\bullet}_{V,N})) \\ d_W(\text{PD}_i(W^{\bullet}_{V,T}), \text{PD}_i(W^{\bullet}_{V,M})) \\ d_W(\text{PD}_i(W^{\bullet}_{V,N}), \text{PD}_i(W^{\bullet}_{V,M})) \end{pmatrix}$
2	tumor cells, necrotic cells, M_1 macrophages, and M_2 macrophages	$\begin{pmatrix} d_B(\text{PD}_i(W^{\bullet}_{V,T}), \text{PD}_i(W^{\bullet}_{V,N})) \\ d_B(\text{PD}_i(W^{\bullet}_{V,T}), \text{PD}_i(W^{\bullet}_{V,M1})) \\ d_B(\text{PD}_i(W^{\bullet}_{V,N}), \text{PD}_i(W^{\bullet}_{V,M2})) \\ d_B(\text{PD}_i(W^{\bullet}_{V,N}), \text{PD}_i(W^{\bullet}_{V,M1})) \\ d_B(\text{PD}_i(W^{\bullet}_{V,N}), \text{PD}_i(W^{\bullet}_{V,M2})) \\ d_B(\text{PD}_i(W^{\bullet}_{V,M1}), \text{PD}_i(W^{\bullet}_{V,M2})) \\ d_W(\text{PD}_i(W^{\bullet}_{V,T}), \text{PD}_i(W^{\bullet}_{V,N})) \\ d_W(\text{PD}_i(W^{\bullet}_{V,T}), \text{PD}_i(W^{\bullet}_{V,M1})) \\ d_W(\text{PD}_i(W^{\bullet}_{V,N}), \text{PD}_i(W^{\bullet}_{V,M2})) \\ d_W(\text{PD}_i(W^{\bullet}_{V,N}), \text{PD}_i(W^{\bullet}_{V,M1})) \\ d_W(\text{PD}_i(W^{\bullet}_{V,N}), \text{PD}_i(W^{\bullet}_{V,M2})) \\ d_W(\text{PD}_i(W^{\bullet}_{V,M1}), \text{PD}_i(W^{\bullet}_{V,M2})) \end{pmatrix}$

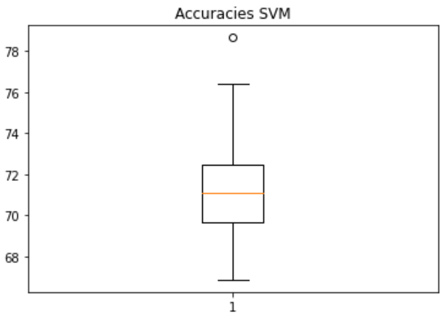

Fig. 2 SVM accuracy on multispecies witness PH distance vectors. We perform SVM analysis to predict the dominant macrophage subtype as described in main-text Section 5.1 on our witness feature vectors which we create as described in main-text Section 5.2 while distinguishing between M_1 and M_2 macrophages in the analysis. We present the accuracies of the SVM for 100 randomized subsets of unseen data.

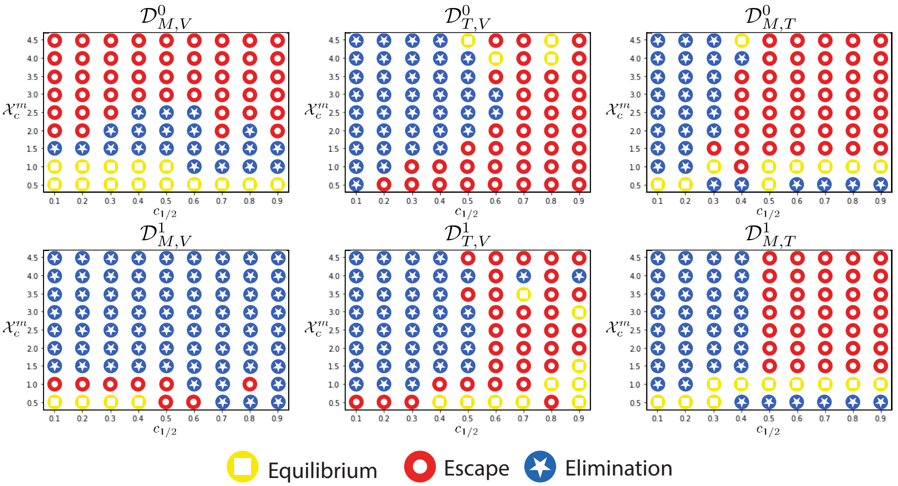

Fig. 3 Classification of qualitative behavior using Dowker persistence images. We perform clustering to infer qualitative behavior regimes as described in main-text Section 5.2 using Dowker persistence images which we create as described in main-text Section 5.1. We present clustering results of each combination of tumor cells, macrophages (without knowledge of phenotype), and blood vessels in dimensions 0 and 1.

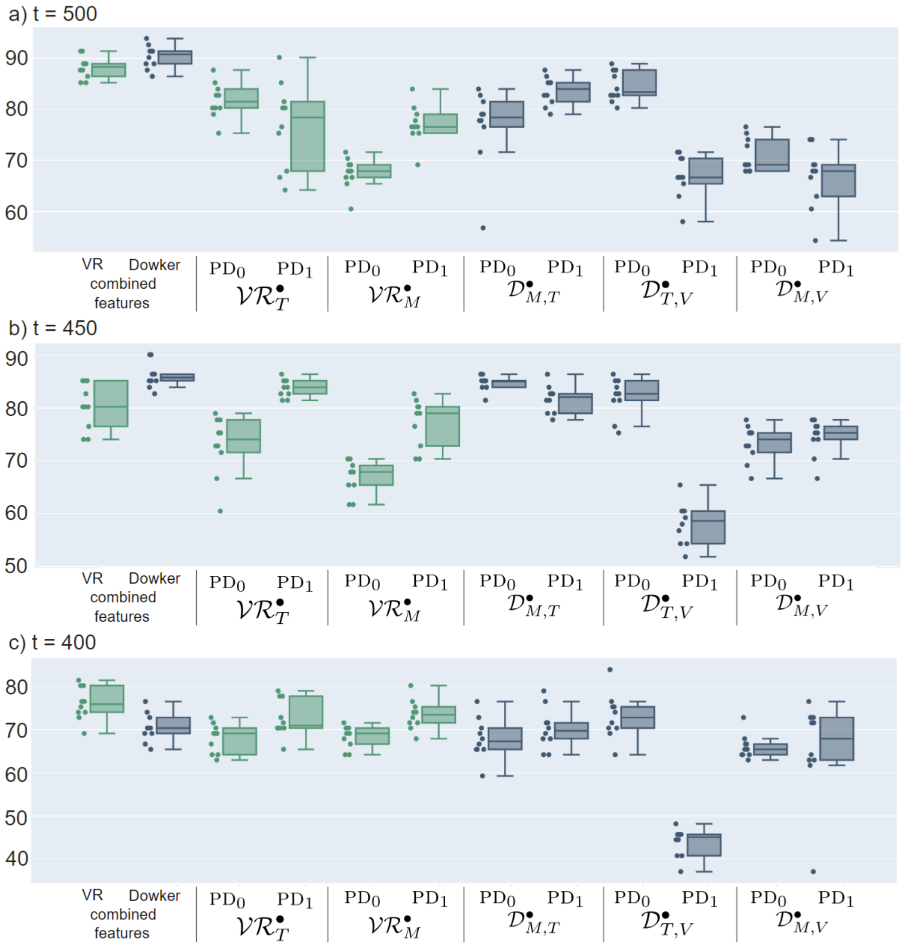

Fig. 4 Classification accuracies of SVMs trained on Vietoris-Rips features (green) and Dowker features (navy) at different time points $t = 500, 450, 400$ show that the performance of the models depend on the time points. At each time point, there are $162 = 2 \times 9 \times 9$ point clouds generated from 2 realizations of 9×9 parameter combinations. As the time point progresses, both SVMs trained on Vietoris-Rips and Dowker features improve their performances, with SVMs trained on the Dowker features experiencing larger improvements. **a** At endpoint $t = 500$, SVMs trained on concatenated Vietoris-Rips features and SVMs trained on concatenated Dowker features perform comparably. There were 74 point clouds labeled 0 (M_1 dominant) and 88 point clouds labeled 1 (M_2 dominant). **b** At $t = 450$, SVMs trained on concatenated Dowker features perform better than SVMs trained on concatenated Vietoris-Rips features. There were 70 point clouds labeled 0 and 92 point clouds labeled 1. **c** At $t = 400$, SVMs trained on concatenated Vietoris-Rips features perform better than SVMs trained on concatenated Dowker features. There were 53 point clouds labeled 0 and 109 point clouds labeled 1.

References

Bonabeau E (2002) Agent-based modeling: Methods and techniques for simulating human systems. Proceedings of the National Academy of Sciences of the United States of America 99 Suppl 3:7280–7. https://doi.org/10.1073/pnas.082080899

Bull JA, Byrne HM (2023) Quantification of spatial and phenotypic heterogeneity in an agent-based model of tumour-macrophage interactions. PLOS Computational Biology 3(19):e1010,994

Dunn GP, Old LJ, Schreiber RD (2004) The three es of cancer immunoediting. Annual Review of Immunology 22(1):329–360. https://doi.org/10.1146/annurev.immunol.22.012703.104803, pMID: 15032581
